# No Evidence for Activity Adjustment in Response to Increased Density in *Daphnia magna*

**DOI:** 10.1371/journal.pone.0144759

**Published:** 2015-12-09

**Authors:** Laura Sereni, Sigurd Einum

**Affiliations:** Centre for Biodiversity Dynamics, Department of Biology, NTNU, Norwegian University of Science and Technology, Realfagbygget, NO-7491 Trondheim, Norway; Evolutionary Biology Centre (EBC), Uppsala University, SWEDEN

## Abstract

Increased population density may lead to a decrease in energy available for growth and reproduction via effects on the activity level of individuals. Whilst this may be of particular importance for organisms that compete for defendable resources and/or have a high frequency of social interactions, it is less obvious how individual activity should covary with population density when food resources are not defendable or direct interactions among individuals are negligible. Based on observations that there is a general negative relationship between population density and metabolism it has been suggested that organisms actively reduce activity under increased density to accommodate an expected decrease in food availability. However, in the absence of direct activity measurements the validity of this hypothesis is unclear. Here we test for such anticipatory adjustments of activity levels in the planktonic cladoceran *Daphnia magna* Straus, a filter feeder whose food resources are not defendable, meaning that density responses can be evaluated in the absence of direct interactions. We tested for changes in activity in response to two separate density cues, one being the direct physical and visual stimuli resulting from being in the vicinity of conspecifics (‘direct density experiment’), and the other being the detection of olfactory cues in their environment (‘olfactory cue experiment’). Ten genetically distinct clones were used to evaluate the potential for genetic variation in these responses. Our measures of activity were highly repeatable, and there was significant variation in activity among clones. Furthermore, this clonal variation was consistent in the ‘direct density’ and ‘olfactory cue’ experiments. The estimated evolvability of the trait (1.3–3.2%) was within the range typically observed in behavioural traits. However, there was no indication that the activity level of individuals respond to population density, either directly to actual density or to olfactory cues representing high density. In this case, the energetic influence of density on population dynamics is sufficiently described by effects on per capita resource availability.

## Introduction

Population regulation by density dependence commonly arises through effects of density on energy that can be allocated to growth and reproduction [1, 2]. Increased population density may lead to a decrease in this component of the energy budget through two main mechanisms. First, for a given amount of food, increased population density will inevitably lead to a reduced *per capita* food availability due to intraspecific competition. A second, but less scrutinised mechanism, is how changes in density affect the amount of energy spent on activity. Increased density may lead to a higher frequency of social interactions among individuals, and this may be accompanied by increased energy spent on activity. This may be particularly relevant for organisms with defendable resources such as feeding territories [3]. However, it is less obvious how energy spent on activity depends on population density when food resources are not defendable and direct interactions among individuals are absent or negligible.

One area of research that has received much theoretical and empirical attention in population ecology is the relationship between density and dispersal. Existing literature suggests a general positive relationship between density and dispersal [4, 5, 6], although negative relationships have also been found [7]. Selective advantages of positive density-dependent dispersal, or more generally movement away from patches of high density, may include both reduced competition [8, 9] as well as avoidance of kin competition and inbreeding [10]. However, selective advantages of adjusting levels of movement activity as a response to density within areas of a more homogeneous distribution of resources are less obvious. One hypothesis of relevance for this stems from a recent review by DeLong et al. [11]. Based on the observation that population density and metabolism are negatively related, and that this relationship stands even in the absence of food, DeLong et al. suggest that, in high density environments, organisms might reduce activity. Since organisms in such experiments do not receive any direct cues regarding the effect of density on food availability, this entails that they are capable of showing an adaptive anticipatory response to ‘budget’ for an expected future reduction in food availability. From a theoretical perspective one might challenge this hypothesis based on the expectation that behavioural adjustments to food availability can be rapid. If so, activity should respond directly to current food abundance/quality, rather than to predicted future conditions. However, this assumes that organisms are able to obtain accurate information on current feeding conditions in terms of their effect on physiological state (i.e. including both food abundance and quality). Thus, one might argue that organisms are limited in their abilities to evaluate current conditions because the physiological state is a result of cumulative processes, and hence time lags between the change in food availability and change in physiological state can be expected. Nevertheless, in the absence of direct activity measurements the validity of the hypothesis remains unknown.

Here we test this hypothesis by measuring activity levels across population densities in the absence of food in the planktonic cladoceran *Daphnia magna*. This species is an ideal organism in which to evaluate density ecology in the absence of direct interactions because it mainly consumes algae and bacteria, i.e. food resources that are not defendable. Furthermore, their swimming activity is known to respond to other environmental variables such as light, food abundance and the presence of predators, suggesting a potential for adaptive activity responses to environmental cues [12, 13]. An additional advantage of using *Daphnia* is that under favourable conditions their reproduction occurs through parthenogenesis, such that any activity related to mating can be disregarded. Finally, the negative relationship between density and metabolism in the absence of food presented by DeLong et al. [11] include data from *Daphnia*, making them highly relevant from a comparative perspective. Given that the swimming behaviour of *Daphnia* is influenced by chemical substances released by conspecifics and predators [14, 15], two separate cues may be responsible for density related changes in activity. One being the direct physical and visual effect of being in the vicinity of a conspecific, and the other being detection of olfactory cues in their medium. Thus, two separate experiments were performed to assess these mechanisms. A set of 10 distinct clones were used to evaluate the potential for genetic variation in the response to density.

## Materials and Methods

### Study animals and husbandry

*Daphnia magna* reproduce by ameiotic parthenogenesis throughout the growing season, and sexual reproduction occurs only under deteriorating environmental conditions, resulting in sexual resting stage eggs called ephippia. Such ephippia were collected in a pond (area 1.0 ha, 1m maximum depth) at Værøy Island, northern Norway (67.687°N, 12.672°E) and hatched in December 2014. No specific permissions were required for this collection, as it was performed on public land and no regulations exist for collection of zooplankton, and the study did not involve any endangered or protected species. Ten clonal lineages were cultured at 17°C with a 16L:8D photoperiod in 250 mL jars containing a modified ADaM medium [16] (SeO_2_ concentration reduced by 50%). Density per jar was kept at five adults or ca. 30 juveniles. These were fed three times a week with Shellfish Diet 1800 (Reed Mariculture Inc, USA) at a final concentration of 4x10^5^ cells ml^-1^, corresponding to 6.8 mg C l^-1^. Medium was exchanged weekly. We obtained juveniles of the F3 generation (10–15 per clone) and reared these under the same light, food and temperature conditions until they reached the adult stage. The day before an experimental trial we isolated adult females from each clone and placed them individually into 30mL vials, fed with Shellfish Diet and kept them there overnight. All individuals were rinsed in ADaM to remove food particles prior to their introduction into the experimental arenas. Ethical approval is not required for performing experiments with *Daphnia*.

### Experimental set-up

Swimming activity was measured in experimental arenas consisting of petri dishes (ø54 mm, 27 mm height, filled with 10 mL ADaM) that were kept at 17°C. The shallow depth (1.5 cm) restricted movement to be primarily in the horizontal dimension, allowing for two-dimensional recording of movement. The arenas were covered with white tape on the outer wall to prevent visual signals among them. Ten arenas were placed at fixed positions (i.e. same positions for each trial) on each of two LED light tables (LED tracing board A4, HUION inc., maximum light intensity) which did not influence the temperature of the medium. The activity of daphnia was recorded with two overhead video cameras (Panasonic^®^HC-V550, shutter 1/250, iris 2.4dB) that were mounted on tripods. The complete setup was covered with a dark blanket to prevent disturbance from changes in light or personnel movement within the room.

### Activity measurement period

We measured activity as the mean swimming velocity (cm s^-1^) over a pre-determined period. Pilot data showed high temporal variation in mean activity when measured over short time intervals (e.g. 5 min). Thus, we calculated mean individual velocity from two 90 min periods (separated by 90 min) for each trial in both experiments (direct density experiment and olfactory cue experiment, see below). The first recording started five minutes after the animals were introduced into the arenas. One trial (i.e. 20 replicate arenas) was conducted per day, and recording always occurred between 10:00 and 14:00. To evaluate the repeatability of the activity measurements we took the mean velocity of the two recordings for each replicate arena and tested for a correlation between these. This was done separately for the two experiments.

### Experimental design, direct density experiment

To test if activity depended on experienced density, 10 trials were performed, and each trial contained one low (one individual) and one high (four individuals) density arena for each of the 10 clones. The high density treatment (400 animals L^-1^) equated to a density which has been shown to induce phenotypic responses in *D*. *magna* (e.g. increased ephippia production, decrease in body size and age at maturation, [17]). To avoid pseudoreplication in the high density replicates, the mean swimming activity of the four individuals was calculated and used in statistical analyses. For all replicates we used the mean activity of the two 90min recordings in statistical analyses.

### Experimental design, olfactory cue experiment

To test if olfactory cues from high population density influenced activity, ADaM from crowded conditions were made by keeping a high density (> 60 individuals L^-1^) of *D*. *magna* (same clones as those tested) in an aquarium and fed with Shellfish Diet for one week (hereafter crowded medium). Control medium was produced in the same way but without *Daphnia*. At the end of the exposure period, crowded and control medium was filtered (0.5μm) to remove algae and other particles. For each trial, five random arenas on each light table were filled with 10mL of crowded medium and the five other with 10mL of control medium. Three trials were conducted, with each containing one individual from each clone in each treatment, yielding a total of 60 replicates (30 of each treatment, six of each clone). As for the direct density experiment, we used the mean activity of the two 90min recordings in statistical analyses for all replicates. Although some olfactory cues would appear in the control treatments during the observational period, its concentration should be negligible compared to what was present in the crowded medium.

### Video analyses

Videos were analysed with EthoVision ^®^XT 10 software (Noldus inc.), which calculated the velocity (in cm s^-1^) travelled by the center point of each animal at a frequency of two samples s^-1^. The detection settings used were: dynamic subtraction image, dark contrast 4–255, size detection 40–100 pixels, no dilution, and no erosion.

### Body size measurements

At the end of each trial all individuals were photographed under a dissecting microscope and body lengths were measured from the top of the head to the base of the tail spine using ImageJ. For the high density replicates the mean body length of the four individuals was used in the analyses. Mean body lengths for the direct density experiment was 3.0mm (0.5 SD) for both treatments, and for the olfactory cue experiment 3.2mm (0.3 SD) for both treatments.

### Statistical analysis

All statistical analyses were conducted with the software R, version 3.1.2 [18], and the results from the two experiments were analysed separately. Linear mixed models were implemented using the *lmer* function from the *lme4* package with Gaussian error distribution [19]. For the direct density experiment, random effects of the full model were trial, arena position and clonal identity (all factors), whereas fixed effects were body length (covariate) and density treatment (factor). The following interaction terms were included: clonal identity × density, and body length × density. For the olfactory cue experiment a similar model structure was employed, except that trial was modelled as a fixed factor due to its few levels (three). For both experiments, model selection was performed using a backwards selection procedure, starting with the random effects and comparing simplified models using likelihood ratio tests [20]. Model simplification proceeded until no further terms could be removed without causing a significant (P < 0.05) decrease in the log-likelihoods (calculated based on REML for deletion of random terms and based on ML for fixed terms). For both experiments we also calculated the broad sense evolvability of activity as *e*_*μ*_ = 100*V*_*a*_/*m* ^*2*^, where *V*_*a*_ is the broad sense genetic variance estimated from the final mixed models described above, and *m* is the mean activity. Such mean scale evolvabilities give the expected percentage change in the trait per unit strength of selection, and are suggested to be more useful for comparative studies of evolutionary potential than heritability estimates [21].

Given that the same set of clones was used in direct and olfactory experiments, we also investigated if any differences observed among clones were consistent in the two types of environment (i.e. real and perceived density). This was achieved by extracting the best linear unbiased predicted values (BLUPs) for each clone from the final model for both experiments using the *ranef* function. We then tested whether clone-specific activity estimates were correlated between the two experiments.

## Results

### Repeatability between recordings

**Fig 1:**
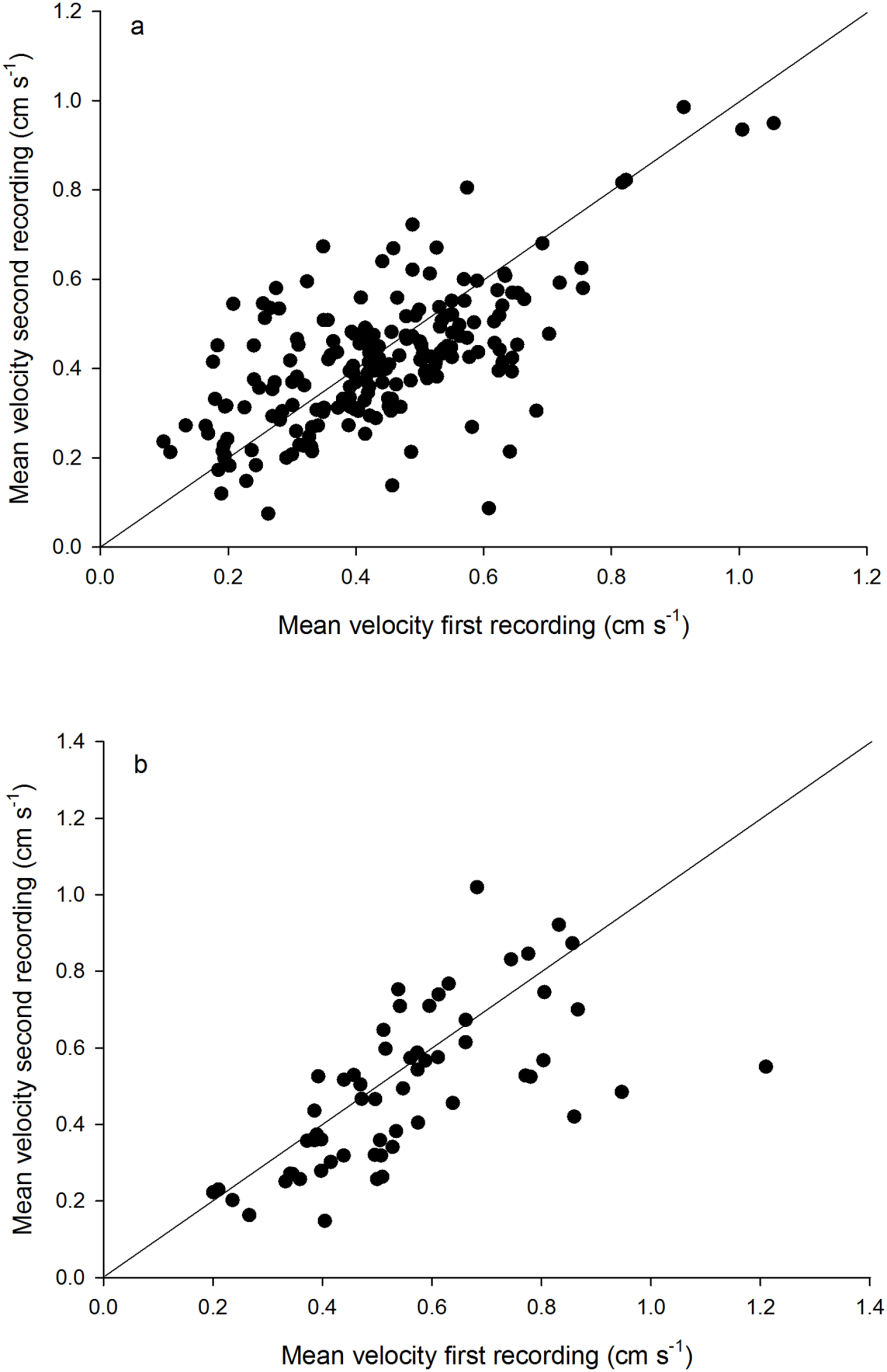
Mean swimming velocity of *Daphnia magna* during two separate recording periods separated by 90 minutes in experiments testing for effects of (a) direct density and (b) olfactory cues. Each data point represents the mean from one replicate arena, and data from different treatments are pooled. Lines represent 1:1 relationships, and *r* ^*2*^-values for the relationships for (a) and (b) are 0.41 and 0.42, respectively.

For both experiments there was a significant positive correlation of the mean activity during the two recordings (Fig 1), with 41–42% of the variation in the second recording being explained by variation in the first recording. The absolute mean velocities of the two recordings were also similar, being 0.44 vs. 0. 42 cm s^-1^ and 0.55 vs. 0.49 cm s^-1^ for the direct density and olfactory cue experiments, respectively.

### Direct density experiment

**Fig 2:**
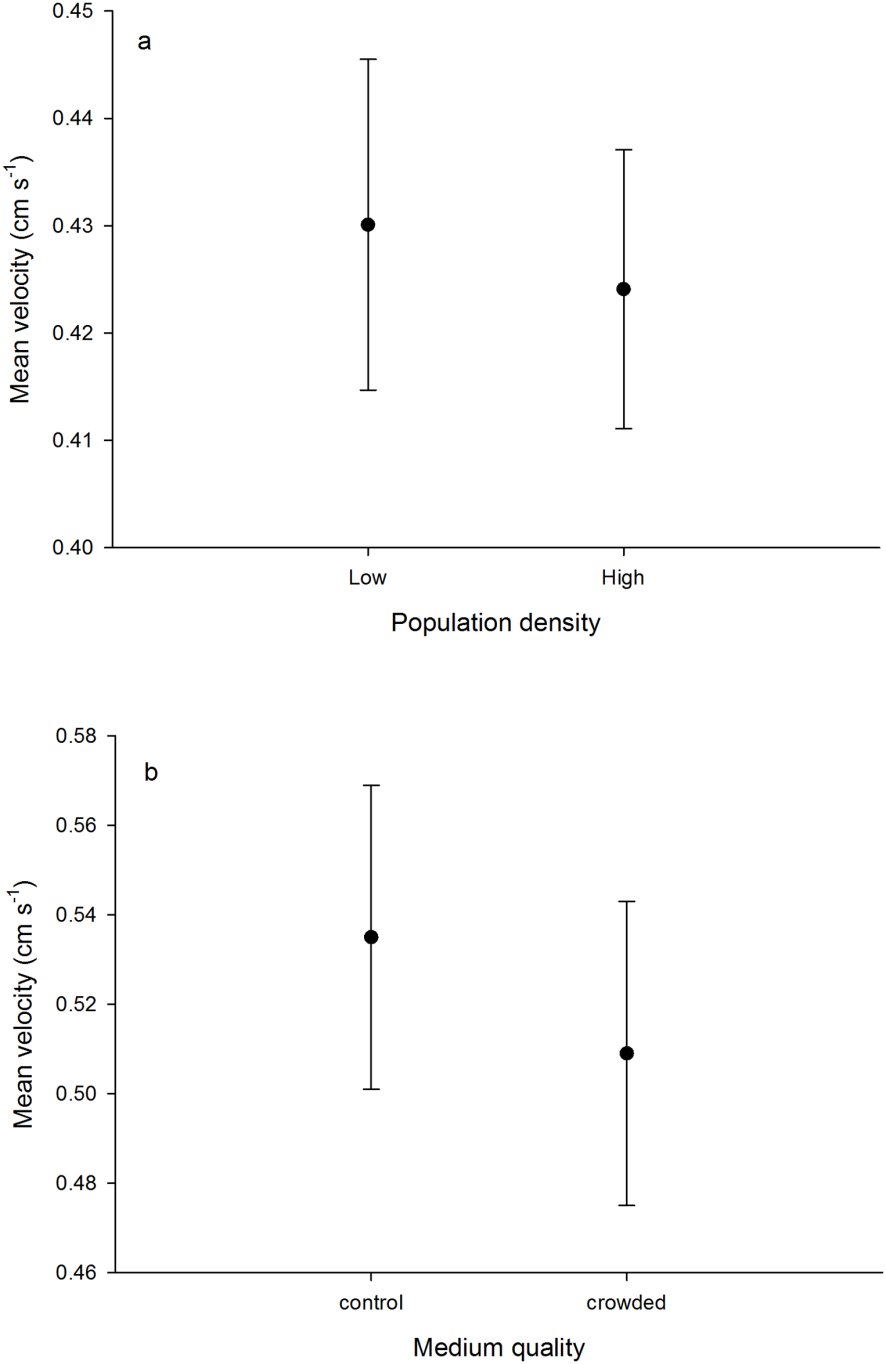
Mean swimming velocity (±SE) of *Daphnia magna* when measured under (a) low (1 individual arena^-1^) or high (4 individuals arena^-1^) population density, and (b) control medium or medium from a crowded environment.

In the model selection of the direct density experiment both the interaction between clone and density (log-likelihood contrast, P = 0.772) as well as the random effect of arena (P = 0.991) could be removed. However, neither trial nor clone identity could be removed (P < 0.001 for both). There was no interaction between body length and density (P = 0.732). Furthermore, mean individual activity was very similar between the two density treatments (Fig 2a), and thus the density term could be removed from the final model (P = 0.746). Mean body length was positively related to mean velocity (slope 0.10 cm s^-1^ mm^-1^ ± 0.04 SE) and could not be removed from the final model (P = 0.006).

### Olfactory cue experiment

In the model selection of the olfactory cues experiment the interaction between clone and treatment was removed from the final model (P = 0.907), as could the main effect of arena identity (P = 0.463), but not clone identity (P = 0.005). For the fixed effects, the main effects of trial and treatment (Fig 2b) as well as the interaction between body length and treatment were removed (P = 0.794, 0.469 and 0.249, respectively). Finally, body length was removed as a main effect, although there was a tendency for velocity to increase with increasing body length with a slope similar to that estimated in the density treatment experiment (P = 0.073, estimated slope in model containing body length 0.13 ± 0.07 SE).

### Clonal effect

**Fig 3:**
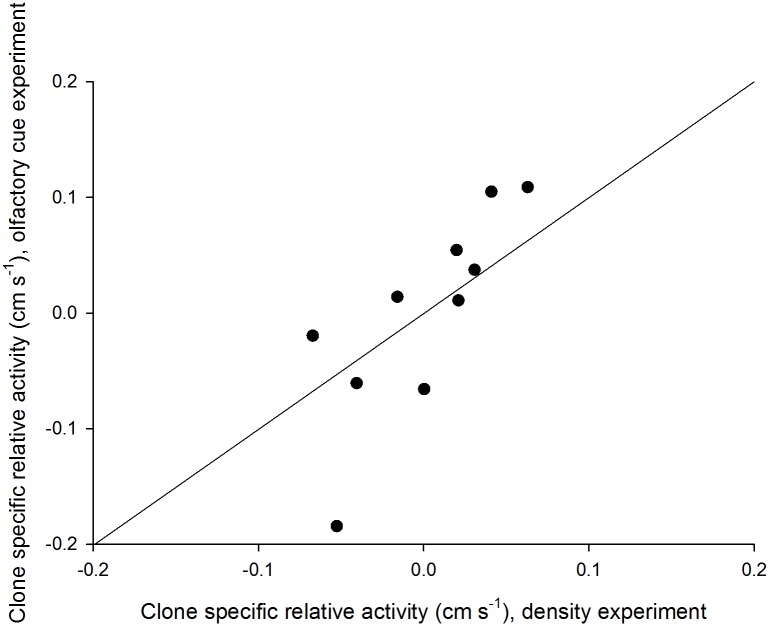
Relative swimming activity of 10 clones of *Daphnia magna* estimated as BLUPs from the best mixed linear models explaining variation in two separate experiments (direct density and olfactory cue). Each data point is calculated based on 20 and six replicates per clone for the density and olfactory cue experiments, respectively. Line represents the 1:1 relationship, and the *r* ^*2*^ for the relationship is 0.62 (significance of correlation P = 0.007).

The clone specific activity patterns were consistent between the two experiments, as shown by a significant positive relationship between their BLUPs (Fig 3). The mean of the two BLUPs for any given clone ranged from -0.12 to 0.09 cm s^-1^. Since the overall mean velocity from both experiments was 0.45 cm s^-1^, this corresponds to a deviation from the mean ranging from -27% to +20%. Evolvabilities were estimated to be 1.3% and 3.2% for the direct density and olfactory cue experiments, respectively.

## Discussion

In the present study we measured levels of activity in 10 clones of *D*. *magna*. The mean velocity observed was ca. 0.4–0.5 cm s^-1^ (ca. 2 times the body length per second), which is similar to estimates in a previous study of *D*. *magna* measured in two dimensions [22]. Our measures of activity were repeatable when comparing values obtained during two different time periods within a day. Furthermore, significant clonal variation was observed in both the direct density and olfactory cue experiments. Finally, the relative activity level of each clone was positively correlated between the two experiments. Thus, our method for measuring activity, which assumes that activity is well represented by swimming speed, yielded highly repeatable data that were able to accurately describe sources of variation. Despite the repeatability of these measurements, our data show no indication that *D*. *magna* adjust their level of activity to population density, neither as a direct response to density nor to olfactory cues representative of a high density environment.

We are not aware of other studies testing for effects of density *per se* on levels of activity for *Daphnia*. In fact, studies that investigate the relationship between density and activity in the absence of food are rare, making it difficult to generalize upon the effects of density beyond the influence that it has on per capita resource availability. One exception is the study by Kuefler et al. [23], where the swimming velocity of rotifers was observed to increase in high density conditions, irrespective of the presence/absence of food. Although we cannot identify why our results differ from Kuefler et al., it is important to note that neither study reports *decreased* activity in response to increased population density, as hypothesised by DeLong et al. [11]. This lends some support to the hypothesis that reported declines in metabolic rate with increasing density are primarily due to reduced food availability and corresponding decreases in specific dynamic action (i.e. energy expenditure due to the costs of processing food, e.g. [24]), and not population density *per se*. However, further empirical studies including direct measurements of metabolic rate seem required to resolve these issues.

Although differences in swimming activity have been documented among *Daphnia* genotypes from different populations [25], the significant within-population variation in swimming activity of different clones reported here, is to our knowledge, novel. This finding is corroborated by a handful of studies on other organisms that also demonstrate genetic within-population variation in levels of activity (e.g. snakes [26], butterflies [27], *Drosophila* [28]). The estimated evolvabilities were within the range typically observed in behavioural traits (Table 1 in [21]) and demonstrate the potential for activity levels to respond to selection. Possible sources of such selection include competition for mates, food and other restricted resources as well as risks of predation. In addition, increased activity may lead to increased dispersal. Indeed, studies on butterflies and drosophila have found a strong correlation between dispersal traits and general activity [27, 28]. Thus, any selection for or against dispersal could cause a correlated evolutionary effect on general levels of activity and *vice versa*.

In contrast to these organisms where general levels of activity may be correlated with and evolve together with levels of dispersal, *Daphnia* inhabit isolated ponds and lakes, and any dispersal among populations can only occur *via* the passive transport of resting eggs. Furthermore, their largely parthenogenetic mode of reproduction means that there should be relatively less selection on activity arising through effects on mating success. Thus, variation in levels of activity in *Daphnia* may be largely explained by selection resulting from variation in food and predator abundance. This makes them an ideal model species for empirical studies of how these selective forces shape genetic variation in activity within- and across- populations. An optimality model developed specifically for fish larvae that incorporates the risk of predation predicts decreased activity with increasing food abundance [29]. Furthermore, even in the absence of predation costs of activity, modelling predicts that for given food abundance there is a bioenergetically optimal activity metabolism, with the optimum being highest at intermediate food abundances [30]. Since food abundance (particularly algae quality and quantity) is highly variable throughout the year for *Daphnia* populations, selection pressures with respect to activity may vary accordingly. This may contribute to maintaining the genetic variation in this trait observed in the current study. For predation pressure, most theoretical models assume that increased activity leads to increased predation (e.g. [31, 32, 33]), and this has been confirmed empirically in *Daphnia* [25, 34]. Thus, one may predict the evolution of lower activity of *Daphnia* from populations inhabiting environments characterised by high predation pressure.

## Conclusions

We found *Daphnia* behavioural activity to be genetically variable as shown by consistent differences among clones, but no indication that they alter their levels of activity in response to direct and perceived increases in density. Even if the optimal activity may depend on food abundance [29, 30], and previous studies have shown that organisms may adjust both their metabolism [35] and their activity in response to food abundance [36, 37, 38], population density is not necessarily a consistent predictor of food abundance. In general, both population density and food abundance can show large temporal fluctuations and population density dynamics can lag behind changes in food abundance, and *Daphnia* are no exception [39]. If so, assessed density represents an unreliable cue for adjusting activity levels. Under such circumstances any adjustments of activity may have to rely on direct detection of food abundance rather than population density. However, even this environmental factor does not appear to influence swimming velocity in *Daphnia* [22]. Thus, in this case, the energetic influence of density on population dynamics is sufficiently described by effects on per capita resource availability.

## Supporting Information

S1 Data Data on activity of *Daphnia magna*. (XLSX)
